# Recurrent Germline BRCA2 Gene Mutation in Lithuanian Family

**DOI:** 10.3390/medicina56030119

**Published:** 2020-03-10

**Authors:** Ieva Sadzevičienė, Olga Liaugaudienė, Justinas Besusparis, Jolita Asadauskienė, Ilona Kulikienė, Birutė Brasiūnienė, Rasa Sabaliauskaitė, Sonata Jarmalaitė

**Affiliations:** 1 Institute of Biosciences, Life Sciences Center, Vilnius University, Saulėtekio Avenue 7, LT-10222 Vilnius, Lithuania; sadzeviciene.ieva@gmail.com; 2 Department of Consultative Polyclinic, National Cancer Institute, Santariškių 1, LT-08406 Vilnius, Lithuania; olga.liaugaudiene@gmail.com; 3 Faculty of Medicine, Vilnius University, Čiurlionio, 21 LT-03101 Vilnius, Lithuania; justinas.besusparis@gmail.com; 4 National Center of Pathology, Affiliate of Vilnius University Hospital Santaros Clinics, P. Baublio 5, LT-08406 Vilnius, Lithuania; 5 Department of Medical Oncology, National Cancer Institute, Santariškių 1, LT-08406 Vilnius, Lithuania; jolita.asadauskiene@nvi.lt (J.A.); birute.brasiuniene@nvi.lt (B.B.); 6 Laboratory of Genetic Diagnostic, National Cancer Institute, Santariškių 1, LT-08406 Vilnius, Lithuania; ilona.kulikiene@nvi.lt (I.K.); rasa.sabaliauskaite@nvi.lt (R.S.); 7 Laboratory of Clinical Oncology, National Cancer Institute, Santariškių 1, LT-08406 Vilnius, Lithuania

**Keywords:** breast cancer, *BRCA* testing, *BRCA2*, c.3847_3848delGT

## Abstract

Approximately 10% of all breast cancer (BC) cases are familial and caused by inheritance of mutant *BRCA1*, *BRCA2,* or some other genes from the same DNA reparation pathway. Genetic counseling in families with cancer history is a powerful means for early cancer detection and active risk reduction through preventive interventions. This is the first report of the rare inherited *BRCA2* frameshift-deletion mutation c.3847_3848delGT in one Lithuanian pedigree with the intense familial history of BC. Three *BRCA2*-positive blood relatives with BC of different biological types were identified in this pedigree with the same type mutation. All three cases were diagnosed with advanced stage ductal carcinoma. Markedly, polymorphic cells and numerous mitoses were identified in BC from the cases. Two patients from the family were diagnosed with the triple negative tumors, while one case had early onset of the hormone positive BC. Despite the variation in clinical and biological presentation of BC, all cases showed a good response to conventional treatment. In conclusion, the strong influence of *BRCA2* mutation on the onset of BC of various biological types reveals the complexity of genetic counselling in families with BC history.

## 1. Introduction

Breast cancer (BC) is the most commonly diagnosed malignancy and leading cause of cancer death among women in the world [1]. About two million new BC cases are diagnosed each year worldwide. Approximately 10% of all BC cases are heritable and caused by a germline DNA damage response gene mutation, mainly of the *BRCA1* or the *BRCA2* genes. Both genes (*BRCA1/2*) encode important tumor suppressors involved in the DNA double-strand break (DSB) reparation by homologous recombination [2]. *BRCA1* mutation carriers possess a 55–65% average cumulative risk of breast cancer by age 70, and the risk for *BRCA2* mutation carriers is slightly lower—45–55%. Similarly, the 10-year risk of developing ovarian cancer has been reported to be 12.7% and 6.8% for women carrying *BRCA1* and *BRCA2* mutations, respectively [3]. *BRCA1/2* mutation-positive cases are more frequently diagnosed with the late-stage tumors at a younger age as compared to the sporadic BC. However, under appropriate treatment regimens, including ordinary chemotherapeutic agents, *BRCA1/2* mutation-positive breast cancer patients have similar survival outcomes as in the mutation-negative cases with sporadic tumors [4]. Platinum derivatives as well as anthracyclines, directly or indirectly inducing DSB, are usually used for the treatment of *BRCA1/2* mutation-positive tumors, and currently registered inhibitors of the enzyme poly ADP polymerase (PARP) provide an even better clinical effect through induction of the synthetic lethality effect [5].

Founder mutations that have common ancestral haplotypes predominate in particular populations, including *BRCA2* c.5946del in Ashkenazi Jews or *BRCA2* c.771_775del in the Icelandic population, while other recurrent mutations, including *BRCA2* c.3847_3848del, are also frequent but have not been characterized as true founder mutations up to now. Currently, more than 1800 sporadic and germline *BRCA2* gene mutations are registered in databases [3]. The frameshift-deletion mutation c.3847_3848delGT is predominant among Caucasian (2%), with 0.4% frequency found in Jews, but is almost undetectable in Asian, Hispanic/Latino, or African American groups [6]. In the European population, and particularly in the Baltic region, the overall frequency of this mutation is extremely rare.

*BRCA1* mutations usually are associated with a particular biological BC subtype, while no such associations were reported for the *BRCA2*-positive cases. *BRCA1*-mutated tumors more likely are estrogen receptor (ER) and progesterone receptor (PR) negative and show no overexpression of HER2 [7], while this triple-negative phenotype is less frequent among *BRCA2*-mutated families. Among triple-negative BC cases, the frequency of germline *BRCA2* pathogenic variants ranges from 3% to 17% [8,9], and thus data on clinical outcomes and response to conventional treatment regimens are extremely scarce. We are the first to report the inherited *BRCA2* frameshift-deletion mutation c.3847_3848delGT in the Lithuanian population, identified in three blood relatives with BC of hormone negative or positive phenotype with accompanying pathologies.

## 2. Materials and Methods

### 2.1. Genetic Counseling

**Figure 1:**
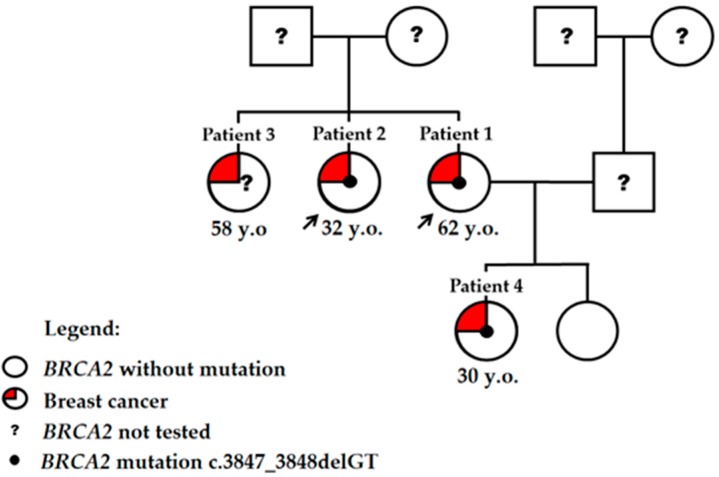
Pedigree of individuals with *BRCA2* c.3847_3848delGT mutation. Patients described in details are indicated by the arrow. Three sisters were diagnosed with breast cancer, while genetic testing was performed for two of them. Two daughters of the proband were tested for *BRCA2* mutation, and one was mutation and cancer-positive. Y.o.: Age at diagnosis is indicated.

In 2017, a 62-year-old woman (Patient 1) was referred to a clinical geneticist with suspected hereditary breast and ovarian cancer (HBOC), and genetic testing for *BRCA1/2* mutations was performed with a positive finding. The hereditary BC pedigree of all of the family is shown in Figure 1. All subjects gave their informed consent for inclusion before they participated in the study. Bioethics approval was obtained from the Vilnius Regional Biomedical Ethics Committee (158200-18-989-493).

### 2.2. DNA Extraction and Mutation Analysis

Blood samples were collected into ethylenediaminetetraacetic acid (EDTA) blood collection tubes according to standard procedures. Tubes were centrifuged at 2300 rpm for 10 min at room temperature to fractionate the whole blood. This separated blood into an upper plasma layer, a lower red blood cell (RBC) layer, and a thin inter-face containing the white blood cells (WBCs). Then, 250 μL of WBCs were collected into a new collection tube and stored at −20 °C or used for DNA purification. DNA extraction was done by a fully automated robotic QIAcube system workstation using QIAamp DNA Blood Mini Kit (Qiagen, Hilden, Germany). DNA concentration and purity were determined using the NanoDrop 1000 spectrophotometer (Thermo Scientific, Wilmington, DE, USA). First, the 6 most common *BRCA* genes mutation in local population were checked, including *BRCA1*: c.4035delA, c.5266dupC, c.181T>G and c.1687C>T; *BRCA2*: c.658_659delGT and c.8572C>T. For this, real-time PCR reactions were done with TaqMan™ Universal Master Mix II, No UNG (Applied Biosystems part of Thermo Fisher Scientific, Vilnius, Lithuania) and TaqMan™ SNP Genotyping Assays (Applied Biosystems part of Thermo Fisher Scientific, Paisley, UK), including wild-type and positive controls. All QPCR reactions were performed according to the manufacturer’s protocol using the Applied Biosystems™ 7500 Real-Time PCR System (Applied Biosystems part of Thermo Fisher Scientific, Foster, CA, USA). After the exclusion of the most common *BRCA1* and *BRCA2* mutations, a next generation sequencing (NGS) was performed. DNA concentration was determined on a Qubit™ 2.0 Fluorimeter using the Qubit™ dsDNA BR Assay Kit (Invitrogen, part of Thermo Fisher Scientific, Eugene, OR, USA). For library preparation, a set of reagents from the Ion AmpliSeq™ Library Kit 2.0 (Life Technologies, Carlsbad, CA, USA) and OncomineTM *BRCA* Research assay was used to amplify the coding regions of the *BRCA1* and *BRCA2* genes under conditions provided by the manufacturer’s protocol. Library concentrations were determined with the Ion Library TaqMan™ Quantification Kit (Life Technologies, Vilnius, Lithuania). Libraries in equal concentration were pooled together for automatic template preparation on the Ion Torrent™ Ion Chef™ Instrument using The Ion 520™ and Ion 530™ KitChef (Life Technologies, Carlsbad, CA, USA). Sequencing was performed on the Ion Torrent™ Ion S5™ (Life Technologies, Carlsbad, CA, USA) system sequencer.

### 2.3. Immunohistochemistry

The surgical specimens were formalin fixed, and gross inspection was performed by slicing the specimens at 5 mm intervals. Representative tumor areas, including margin designation, were selected for further processing. Immunohistochemistry (IHC) was performed by using formalin-fixed paraffin-embedded full-face sections, which were cut 3 μm thick and mounted on positively charged slides. A Roche Ventana BenchMark ULTRA automated staining system (Ventana Medical Systems, Tucson, AZ, USA) was used to perform the IHC staining. ER, PR, HER2, and Ki67 were detected using the ultraView Universal DAB Detection kit. IHC was performed using ready-to-use antibodies for ER, PR, and HER2 (SP1, 1E2, 4B5, respectively, Ventana (Tucson, AZ, USA), Ki67 (MIB-1, DAKO (Glostrup, Denmark), dilution 1:200).

## 3. Case Report

**Figure 2:**
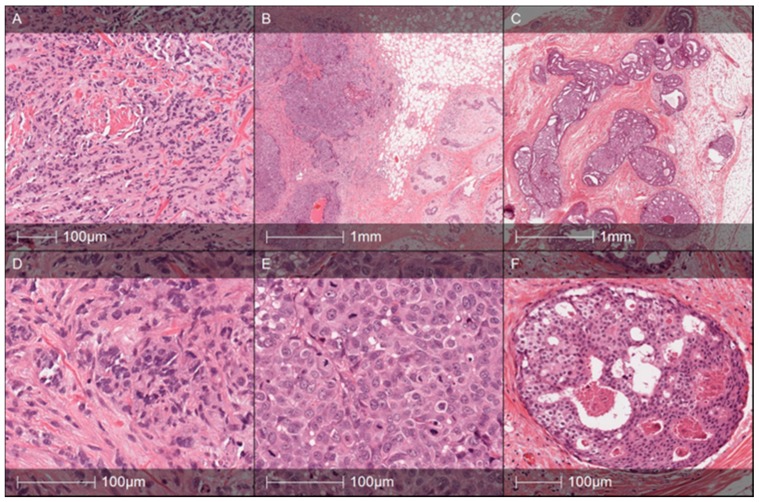
Histology specimens of the proband (age 62) (**A**,**D**), younger sister, Patient 2 (age 32) right breast tumor, year 2008 (**B**,**E**) and left breast lesion in 2014 (**C**,**F**). Proband’s tumor Hematoxylin and Eosin (HE) slides demonstrate poorly differentiated (G3, Nottingham grade 8) ductal carcinoma composed of confluent trabecular structures and tumor sheets without any presence of glandular differentiation (**A**). Tumor cells reveal marked nuclear polymorphism (**D**) and a high mitotic rate (up to 10 mitoses per 10 high-power fields (HPF)). Patient 2′s (year 2008) right breast tumor was diagnosed as high grade ductal carcinoma with solid growth pattern (**B**) and highly polymorphic tumor cells and numerous mitoses (**E**). The tumor was triple negative, expressed high-molecular-weight cytokeratins (CK5 in particular), and was regarded as “basal-like carcinoma”. Note the pushing margin in the periphery of the tumor (**B**). Patient 2′s left breast lesion (year 2014) with ductal carcinoma in situ (DCIS) of high grade: C—multiple spaces involved by cribriform pattern DCIS with some comedo necrosis. A high-power view of the lesion is shown in F. The cells show that nuclear polymorphism and several mitotic figures are evident.

In February 2017, Patient 1 at age 62 was diagnosed with stage III right breast ductal carcinoma cT2N3M0 (Table 1). The tumor was poorly differentiated (Figure 2A–D) and negative for ER, PR, and HER2 receptor expression on IHC, i.e., triple negative breast cancer (TNBC). According to current recommendations for genetic testing of TNBC cases, genetic counseling was suggested for the patient. NGS analysis revealed pathogenic *BRCA2* gene frameshift-deletion mutation c.3847_3848delGT (4075delGT; p.Val1283Lysfs). 

Patient 1 received four cycles of doxorubicin and cyclophosphamide (AC) neoadjuvant chemotherapy in standard doses and schedules and 12 doses of paclitaxel every week. Complete regression was achieved after neoadjuvant chemotherapy. On the computer tomography scan, complete regression was documented for the primary tumor and the lymph nodes with no evidence of distance metastases. In November 2017, based on the patient’s decision, a radical mastectomy was performed. Postsurgical examination revealed complete pathological response after chemotherapy diagnosis ypT0ypN0. Adjuvant radiotherapy to breast scar and regional lymph nodes was performed. Currently, Patient 1 is on active surveillance.

Based on Patient 1′s information, other blood relatives performed the genetic testing. NGS analysis revealed *BRCA2* mutation positivity in the proband’s sister and daughter, both already diagnosed with BC prior to the genetic testing. The older sister of the proband (Patient 3, age 58) was also BC-positive, but no information on genetic status or clinical course is available for this case.

In 2001, the younger sister of the proband (Patient 2) was initially diagnosed with left breast ductal cancer of stage III (pT2N2M0) at age 32. After two cycles of AC neoadjuvant chemotherapy, the patient underwent a left breast quadratectomy and lymphadenectomy, where eight of 11 lymph nodes were metastatic. The tumor was ER and PR-negative, while the HER2 status was not tested at that time. After surgery, the patient received one cycle of AC chemotherapy then adjuvant radiotherapy to her left breast and an additional three cycles of AC chemotherapy.

Seven years later, in 2008, Patient 2 was diagnosed with poorly differentiated (G3) invasive ductal adenocarcinoma of the right breast, pT1N0M0 (Figure 2B–E). She underwent a right breast quadrantectomy and lymph nodes removal; all of them were metastases free. The tumor was subtyped as TNBC. After surgery, the patient received four cycles of EC chemotherapy (epirubicin and cyclosphamide in standard doses and schedules) and adjuvant radiotherapy to the right breast.

After six years, in 2014, carcinoma in situ was detected in her left breast (ER and PR negative), and a left breast mastectomy was performed (Figure 2C–F).

In 2017, three years later, a right breast mastectomy was performed due to the poorly differentiated (G3) lobular carcinoma, pT1N0M0. However, the tumor was ER 100%, PR 90%, with HER2 amplification and Ki67 50%. After the mastectomy, the patient received paclitaxel and trastuzumab in standard doses and schedules. Seven months after chemotherapy, Patient 2 was diagnosed with uterus adenomyosis and intramural leiomyoma, and a hysterectomy was performed.

**Figure 3:**
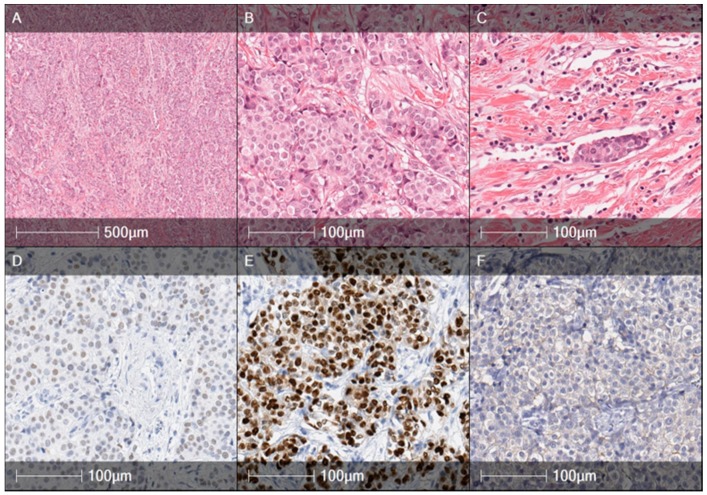
Histology specimens of the proband’s daughter (age 30), right breast tumor, year 2007. Hematoxylin and Eosin (HE) slides (**A**,**B**) illustrate a moderately differentiated (G2) ductal carcinoma composed of trabecular, alveolar structures, and tumor nests. Carcinoma cells revealed an intermediated grade of nuclear polymorphism with mitotic counts up to 8 mitoses/10 HPFs. Intravascular invasion was present (**C**). The tumor was weakly positive for ER + (**D**), strongly positive for PR + (**E**), and negative for HER2 (**F**).

Two daughters of the proband were also involved in the study. One daughter was diagnosed as *BRCA* mutation and cancer-free, while the other daughter (Patient 4) inherited pathogenic *BRCA2* gene mutation and was diagnosed with BC at the age of 30. A diagnosis of moderately differentiated (G2) invasive ductal right breast carcinoma of stage II (pT2N1M0) was registered in 2007 without testing mutational status. The tumor was ER, PR positive, and HER2–negative (Figure 3A–F).

Right breast quadrantectomy and lymph nodes removal were followed by chemotherapy, radiation therapy, and hormone therapy. Seven years later, in 2014, Patient 4 was diagnosed with ductal carcinoma of the right breast and underwent a right breast mastectomy followed by hormone therapy. Soon, ovaries were removed due to marginal changes. Genetic mutation testing, however, was performed only in 2018, based on the TNBC diagnosis of her mother.

## 4. Discussion

*BRCA2* is a tumor suppressor gene located at the 13 chromosome q13.1 locus that covers 84.2 kb of genomic DNA. The gene is composed of 27 exons with the translation site located in exon 2. Over 730 protein-truncating mutations were detected in the *BRCA2* gene, and approximately half the mutations were detected in exon 11, which comprises about 60% of the gene coding region [10]. Although in BC, mutations of *BRCA2* are less common than in *BRCA1*, a high percentage of frameshift mutation is detectable in both genes (*BRCA1*-58%, *BRCA2*-66%). *BRCA2* c.3847_3848delGT (4075delGT, rs80359405) pathogenic mutation causes frameshift and generates mutant p.V1283fs * 2 protein. This type of *BRCA2* mutation is uncommon among the Ashkenazi population [11] but is quite widespread in northern and western Europe, being one of five most frequently observed mutations. In 26 Lithuanian families, Rebbeck et al. [6] described 11 unique mutations, and the five most frequently observed *BRCA2* mutations included c.658_659del (13), c.3847_3848del (4), c.6580dup (1), c.6410del (1) and c.7879A>T (1). In neighboring Poland, only two family cases have been identified with this type of mutation [12]. Wang and colleagues described 0.02 (95% CI 0.00–0.03, *P* = 0.51 for heterogeneity test) overall frequency for *BRCA2* c.3847_3848delGT mutation in Estonian and German populations [10].

In reported pedigree, at least three *BRCA2* c.3847_3848delGT mutation-positive BC cases were identified, suggesting a high penetrance of the mutation. All three cases with the mutation were diagnosed with advanced stage ductal carcinoma at a quite young age (mean 41 year). The age at BC onset varies in *BRCA2* mutation-positive families with the mean age of 47 years [3,13], while in the described family, two out of three *BRCA2* mutation-positive cases were diagnosed at their 30th year. However, both cases with the early onset of the disease were diagnosed with different histological phenotypes of BC, and various pathological presentations were reported during progression of the disease.

Several studies have demonstrated that cancer arising in carriers of mutations in either the *BRCA1* or *BRCA2* genes differs morphologically from sporadic breast cancers [14,15,16,17]. Familial BC cases associated with *BRCA1* mutations usually have a distinct histologic appearance; these tumors are high grade and have exceptionally high mean mitotic counts, a syncytial growth pattern, pushing margins, and confluent necrosis, while no predominant histological subtype of *BRCA2* cases have been reported up to now [15]. Breast carcinomas from *BRCA2* mutation carriers tend to be of a higher grade than sporadic age-matched controls [14]. The majority of *BRCA2* tumors are grade 2/3 and reveal less tubule formation and higher nuclear pleomorphism and higher mitotic rates [17]. In line with this data, almost all reported cases in the described family were of G3 tumors with markedly polymorphic cells and high mitotic counts but without syncytial pattern or confluent necrosis areas. Dite et al. reported that absence of extensive sclerosis, extensive intraductal carcinoma, trabecular/lobular growth patterns, and absence of glandular growth were all independent predictors of breast cancer for their first-degree female relatives and were associated with an approximate doubling of risk for relatives [18]. Interestingly, all tumors in the described family had a predominant trabecular or lobular-like growth pattern with no signs of extensive fibrosis or gland formation. Additionally, the tumor of the younger sister of the proband (Patient 2) had an extensive high grade in situ lesion around the invasive tumor. Importantly, all invasive BC cases had histological signs of continuous pushing borders in the periphery of the tumor, which is also reported as a feature for *BRCA1* tumors [19]. Besides the fact there is no proof of predominant histological subtype of *BRCA2* associated BC, based on our data, it could be speculated that the mutation type of *BRCA2* can affect tumor histological phenotype.

Unlike *BRCA1* mutations carrying BC, which usually are ductal type but have basal-like/medullary carcinoma-like features, triple negative phenotype, and predispose high frequency of ER-negativity (~80%), *BRCA2* mutation carrying tumors usually are characterized by high ER (~65%) and PR (~50%) [14]. In our study, two out of three mutation carriers eventually were diagnosed with TNBC, but one case was diagnosed with a hormone receptor-positive tumor without clear indications for genetic testing or preventive surgery. The other case with TNBC had tumors of various histologies during the course of the disease (ductal G3, DCIS, and lobular G3 breast cancer). However, Honrado et al. [14] suggest that ER+ and /or PR+ tumors without evidence of basal differentiation should be first tested for *BRCA2* mutations in families with a high incidence of breast and ovarian cancer. One of the most important applications of this histopathological data and *BRCA1/2* mutational status correlations is to guide the treatment decisions.

## 5. Concluding Remarks

Despite variation in biological subtype, histology, and onset of the disease in the described family, the response to neoadjuvant and adjuvant treatment was quite good, and that is in agreement with the published data on hereditary BC [4]. A period of seven years passed until the disease progression was reported both for the hormone receptor-positive as well as for the TNBC case of the pedigree. However, the recurrence of the TNBC case was quite complicated with a bilateral manifestation and range from ductal to lobular histology.

The strong influence of *BRCA2* mutation visible in our described family reveals the importance of genetic counseling in families with cancer history. *BRCA2* c.3847_3848delGT mutation evidence in the described family varied at age at onset, biology and pathology of tumors, and course of the disease. Unfortunately, in this family, the late onset-case was the first one consulted by the geneticist, and the extensive familial history of BC was identified retrospectively. Further investigation of *BRCA2* c.3847_3848delGT mutation-positive BC is required for a better understanding of the clinical and the biological profiles of the disease.

## Figures and Tables

**Table 1:** The summary clinicopathologic characteristics of the patients.

Patient	Year	Characteristics
**Patient 1 (proband)**	2017	Stage III (cT2N3M0) right breast ductal carcinoma, grade 3, estrogen receptor (ER)/progesterone receptor (PR)/HER2 negative. Complete response after chemotherapy pT0ypN0.
**Patient 2 (younger sister of the proband)**	2001	Stage III (pT2N2M0), left breast ductal carcinoma, node positive (8 of 11), ER/ PR negative, HER2 status was not tested at that time.
	2008 2014	Right breast ductal carcinoma, grade 3, pT1N0, ER/PR/HER2 negative. Left breast ductal carcinoma in situ, high grade, ER/PR negative.
	2017	Right breast lobular carcinoma, grade 3, node negative, pT1N0M0. ER 100%, PR 90%, HER2 amplified, Ki67 50%.
**Patient 3 (older sister of the proband)**		Invasive breast carcinoma, no information on genetic status or clinical course is available for this case.
**Patient 4 (proband’s daughter)**	2007	Stage II (pT2N1M0) right breast ductal carcinoma, node positive, grade 2, ER/PR +, HER2–negative.
	2014	Right breast ductal carcinoma, no information on immunohistochemistry is available for this case.
